# Evaluating the Impact of COVID-19 Pandemic on Public Interest in Minimally Invasive Surgery: An Infodemiology Study Using Google Trends

**DOI:** 10.7759/cureus.18848

**Published:** 2021-10-18

**Authors:** Nellai Krishnan, Sachit Anand, Gursev Sandlas

**Affiliations:** 1 Pediatric Surgery, All India Institute of Medical Sciences, New Delhi, IND; 2 Pediatric Surgery, Kokilaben Dhirubhai Ambani Hospital and Medical Research Institute, Mumbai, IND

**Keywords:** google trends, minimally invasive surgery, robotic surgery, laparoscopic surgery, covid-2019, infodemiology

## Abstract

Background

The pandemic caused by the coronavirus disease 2019 (COVID-19) has impacted the healthcare system worldwide, leading to the suspension of elective surgeries and a decline in the utilization of minimally invasive surgeries (MIS). However, an objective parameter depicting the degree of decline of MIS is lacking. We aim to indirectly evaluate the impact of the COVID-19 pandemic on the number of MIS performed by the surgeons by evaluating the public interest in MIS using Google Trends.

Methods

A Google Trends search using the string [“laparoscopic” + “minimally invasive” + “robotic surgery”] was performed on June 2, 2021. The monthly relative search volume (RSV) indices were compared with the number of reported COVID-19 cases during the same period.

Results

RSV was highest between August 2018 and February 2020. RSV at the start of the pandemic was 95 but had declined to 51 during the first COVID-19 peak in April 2020 and 80 during the second peak in May 2021.

Conclusion

The monthly RSV related to MIS on Google Trends is a good tool to indirectly estimate the degree of decline in the number of MIS (both laparoscopic and robotic) performed worldwide during the pandemic.

## Introduction

The coronavirus disease 2019 (COVID-19) pandemic has impacted the healthcare system across the globe [1]. Healthcare utilization in terms of both elective and emergency surgical procedures has suffered from this pandemic. Not only these procedures were delayed or canceled but the routine perioperative management posed a fair degree of challenge to the hospital staff [2].

Minimally invasive surgery (MIS) or keyhole surgery has revolutionized the field of surgery. Over the last few years, MIS has been quite popular and has been the “approach of choice” among the patients because of its benefits including small-sized incisions, brief hospital stay, and early recovery [3]. Similar to other surgical subspecialties, the public interest in MIS during the pandemic has declined due to the cancellation of elective surgical procedures [2]. However, the magnitude of the decline is uncertain. There is a paucity of literature on the public interest in MIS during the pandemic.

In this study, we aim to evaluate the impact of the COVID-19 pandemic on public interest in MIS by comparing the Google Trends data on MIS with the number of newly infected COVID-19 cases during the same time period. We hypothesize that the public interest in MIS in terms of the RSV has declined during the course of this pandemic. The relative search volumes (RSV) during different phases of the pandemic can provide an indirect measure of the degree of decline in the minimally invasive surgeries performed by the surgeons.

## Materials and methods

The Google Trends tool was used to extract the data on internet search activities in the context of MIS. The search string [“laparoscopic” + “minimally invasive” + “robotic surgery”] was used to synthesize monthly RSV data from January 2015 to May 2021. The RSV number represents the proportion of popularity of a term relative to the peak popularity during the reference period. RSV values range between 0 and 100. A value of 100 is the peak popularity for the specified search query in that time period. A value of 50 means that the term is half as popular. A value of 0 signifies that there were not enough data for this term. The Google Trends data and new COVID-19 cases were plotted against time and against each other.

The monthly worldwide data on new confirmed COVID-19 cases diagnosed between January 1, 2020 and May 31, 2021 were retrieved from Worldometer (https://www.worldometers.info/coronavirus/) on the 2nd of June, 2021. The Worldometer is an independent digital media company based in the United States comprising developers and researchers across the world [4]. For the COVID-19 data, it retrieves the information from the official reports, directly from governments' communication channels or indirectly through local media sources when deemed reliable, thereby providing timely updates.

This cross-sectional study involved the analysis of information already available on the internet. Since no actual patient contact was established during this study, a clearance from the Institutional Review Board was not required. Two authors (NK and SA) performed the data extraction. Any discrepancies were resolved via discussion with the third author (GS).

## Results

**Figure 1:**
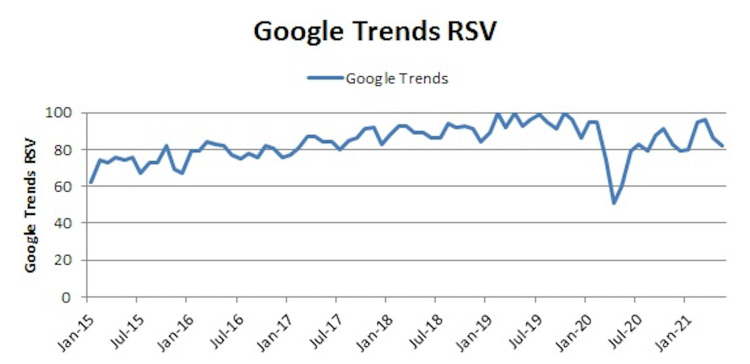
The relative search volumes on Google Trends with respect to time. RSV: relative search volumes.

The Google Trends data showed the highest RSV between August 2018 and February 2020. Following this, there was a sudden and huge decline in the RSV, reaching the lowest in April 2020. Subsequently, there was a gradual rise in the RSV, which peaked in March 2021, declining again till May 2021 (Figure 1).

**Figure 2:**
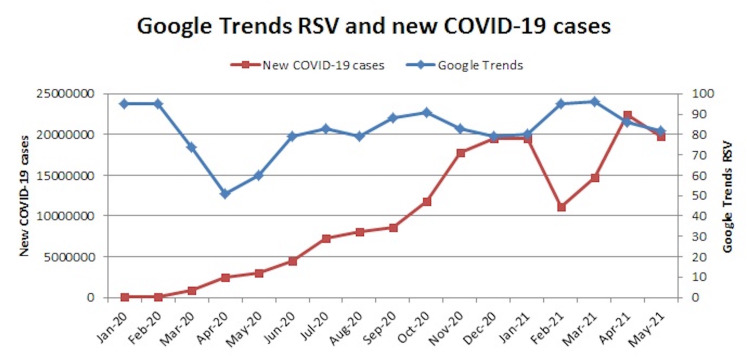
Relative search volumes on Google Trends and newly diagnosed COVID-19 cases with respect to time. RSV: relative search volumes; COVID-19: coronavirus disease 2019.

The number of new COVID-19 cases worldwide increased gradually in the first few months, then rising exponentially and peaked twice; the first peak was in January 2021 and the second peak was in April 2021. The trends of Google searches with respect to the new COVID-19 cases worldwide are plotted in Figure 2.

## Discussion

As of September 30, 2021, there have been almost 234 million confirmed COVID-19 cases worldwide [4]. This has led to the suspension of routine (or elective) surgeries to divert healthcare staff and resources to tackle the pandemic. Similarly, the popularity and usage of minimally invasive surgery (MIS) have also declined during this pandemic. However, an objective parameter depicting the degree of decline in the MIS performed by the surgeons is lacking.

Google Trends is a tool used to study the patterns of search engine queries worldwide [5]. It was implemented in 2004. It allows us to compare the relative search volumes (RSV) based on the popularity of top search queries in Google Search across various regions and languages. The website uses interactive graphs to compare the search volumes of different queries over time. It is a cost-effective means to find the relative public interest on a topic based on the frequency of Google searches performed during a specified time period [6]. It expresses the absolute number of searches relative to the total number of searches over the defined period of interest. Numbers represent search interest relative to the highest point on the chart for the given region and time. These numbers indicate the popularity of the search query at that point in time. Google Trends data make use of millions of users’ searches and have been widely used in the context of health issues [7]. The analysis of the RSV from Google Trends gives information on the extent of public attention and popularity of the search query [8].

In the current era, the internet penetration rate is very high. Patients often “Google” their diseases and treatment modalities. Google has a monopoly in India, with 98.8% of the total search engine market share [9]. A patient who is planned for minimally invasive surgery would often perform an internet search to understand the treatment plan better. So the Google Trends data on MIS can be used as a proxy (indirect measure) for the number of such surgeries being performed at that time. RSV at the start of the pandemic was 95 but had declined to 51 during the first COVID-19 peak in April 2020 and 80 during the second peak in May 2021.

Our analyses demonstrated a decline in the public interest of MIS during the pandemic. During the early stages of the pandemic, due to the risk of aerosol spread of the virus during MIS, surgeons had stopped performing such surgeries. The hospitals worldwide introduced limitations to aerosol-generating procedures (AGPs) for the protection of healthcare professionals (HCPs) and to limit the spread of infection in hospitals. Laparoscopy was deemed to be an AGP due to unavoidable air leaks through the port sites, which could spread the virus [10]. Although the operating surgeon maintains an adequate distance from the patient during robotic surgeries, however, there is a risk of aerosol spread of the virus to the assistant [11]. Moreover, to accommodate the surge of patients with severe COVID-19, all elective surgeries were suspended. These factors led to a decline in all forms of MIS performed worldwide. However, the subsequent studies did not find any scientific evidence to support the use of open surgery over MIS in reducing viral transmission and have suggested few modifications to decrease the theoretical risk of transmission during MIS [12]. This led to the resurgence of MIS after a slight decline. The RSV during the second COVID-19 peak did not decline as much as during the first peak probably due to the adaptations of these modifications in MIS by the surgeons.

There are a few limitations of this study. First, the Google Trends data are an indicator of the popularity of the search terms during a particular time period. Although an indirect measure, it might not represent the actual utilization of MIS. Second, the search data available through Google Trends reflect those with internet access only. Internet penetration is not uniform throughout the globe. In addition, data from other web browsers need to be studied for a comprehensive assessment. Third, only three search terms were used in this study. These might not reflect all the minimally invasive procedures. Finally, we included only search terms used in the English language. The search terms in the other major languages were not accounted for in our study. Despite the above limitations, the present infodemiology study is the first to provide a glimpse of the impact of the COVID-19 pandemic on public interest in MIS.

## Conclusions

The RSV values on Google Trends indicate the popularity and public interest of a particular topic. The present study depicts a decline in the popularity of MIS during the peak increase in COVID-19 cases. These monthly RSV related to MIS on Google Trends can serve as a good tool to indirectly estimate the change in the number of MIS (both laparoscopic and robotic) performed worldwide during the pandemic.

In terms of its implications on the general population, these trends demonstrated that the public interest correlated well with the international guidelines issued during the pandemic. In scenarios of increased strain on the healthcare system, the Google Trends data can be used to check the public interest and preparedness among the general population. However, it is noteworthy that these trends do not depict the treatment-seeking behavior of the patients. Whether the patients, who were planned for MIS during the pandemic, sought their treatment via an open approach or deferred the treatment indefinitely is a subject of further research.

## References

[1] Wang C, Horby PW, Hayden FG, Gao GF (2020). A novel coronavirus outbreak of global health concern. Lancet.

[2] Mohty KM, Lashkari N, Gittings DJ, Bell JA, Stevanovic M, Nicholson LT (2021). Utilizing Google Trends to track online interest in elective hand surgery during the COVID-19 pandemic. Cureus.

[3] Nanavati AJ, Nagral S (2016). Why have we embraced minimally invasive surgery and ignored enhanced recovery after surgery?. J Minim Access Surg.

[4] (2021). Worldometer. https://www.worldometers.info/coronavirus/.

[5] Cohen SA, Zhuang T, Xiao M, Michaud JB, Shapiro L, Kamal RN (2021). Using Google Trends data to track healthcare use for hand osteoarthritis. Cureus.

[6] Thirunavukarasu AJ (2020). Evaluating the mainstream impact of ophthalmological research with Google Trends. [PREPRINT]. Eye (Lond).

[7] Arora VS, McKee M, Stuckler D (2019). Google Trends: opportunities and limitations in health and health policy research. Health Policy.

[8] Venkatesh U, Gandhi PA (2020). Prediction of COVID-19 outbreaks using Google Trends in India: a retrospective analysis. Healthc Inform Res.

[9] Seehra J, Isherwood J, Verma A (2020). Does the risk of SARS-COVID-19 at laparoscopy justify the precautions?. Br J Surg.

[10] Ouzzane A, Colin P (2020). Cost-effective filtrating suction to evacuate surgical smoke in laparoscopic and robotic surgery during the COVID-19 pandemic. Surg Laparosc Endosc Percutan Tech.

[11] Vigneswaran Y, Prachand VN, Posner MC, Matthews JB, Hussain M (2020). What Is the appropriate use of laparoscopy over open procedures in the current COVID-19 climate?. J Gastrointest Surg.

[12] Fong HK, Singh S, Raina JS, Itare VB, Spasova V, Desai R (2021). Alarmingly increased public interest in "Chest Pain" during the COVID-19 pandemic: insights from Google Trends analysis. Cureus.

